# *MRLC* controls apoptotic cell death and functions to regulate epidermal development during planarian regeneration and homeostasis

**DOI:** 10.1111/cpr.13524

**Published:** 2023-06-25

**Authors:** Yujia Sun, Yongding Huang, Zhitai Hao, Shoutao Zhang, Qingnan Tian

**Affiliations:** ^1^ School of Life Sciences Zhengzhou University Zhengzhou Henan China; ^2^ Department of Biochemistry and Molecular Pharmacology New York University, School of Medicine New York USA; ^3^ Longhu Laboratory of Advanced Immunology Zhengzhou Henan China

## Abstract

Adult stem cells (ASCs) are pluripotent cells with the capacity to self‐renew and constantly replace lost cells due to physiological turnover or injury. Understanding the molecular mechanisms of the precise coordination of stem cell proliferation and proper cell fate decision is important to regeneration and organismal homeostasis. The planarian epidermis provides a highly tractable model to study ASC complex dynamic due to the distinct spatiotemporal differentiation stages during lineage development. Here, we identified the myosin regulatory light chain (*MRLC*) homologue in the *Dugesia japonica* transcriptome. We found high expression levels of *MRLC* in wound region during regeneration and also expressed in late epidermal progenitors as an essential regulator of the lineage from neoblasts to mature epidermal cells. We investigated the function of *MRLC* using in situ hybridization, real‐time polymerase chain reaction and double fluorescent and uncovered the potential mechanism. Knockdown of *MRLC* leads to a remarkable increase in cell death, causes severe abnormalities during regeneration and homeostasis and eventually leads to animal death. The global decrease in epidermal cell in *MRLC* RNAi animals induces accelerated epidermal proliferation and differentiation. Additionally, we find that *MRLC* is co‐expressed with *cdc42* and acts cooperatively to control the epidermal lineage development by affecting cell death. Our results uncover an important role of *MRLC*, as an inhibitor of apoptosis, involves in epidermal development.

## Graphical abstract

The planarian adult stem cells have to undergo proliferation and multiple differentiation transition stages to ultimately become mature epidermal cells. Sun et al. identify the myosin regulatory light chain as an essential regulator of the lineage from neoblasts to mature epidermal cells, facilitating proper balance between cell death and stem cell proliferation.

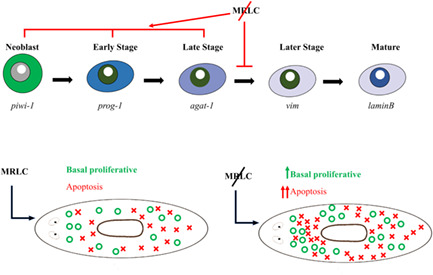

## 1 INTRODUCTION

The epidermis, a stratified epithelium, plays critical roles in animal development and survival. As the outermost tissue of the body, the epidermis can serve as a permeability and structural barrier.^1^, ^2^ In addition to these roles, the epidermis has important physiological functions, which include innate immunity, mechanosensation and wound healing.^1^, ^3^ The epidermis must be renewed by a stem cell population to replenish lost cells and repair the wound. Thus, a precise balance between epidermal progenitor proliferation and differentiation is needed to ensure the formation of a functional epidermis. Previous studies demonstrated that the expression of genes controlling proliferation and differentiation was disrupted in epidermal cancer and skin diseases.^2^, ^4^ However, the mechanisms of epidermal development in vivo have not been thoroughly elucidated.

Planarians are regarded as an excellent model system well suited for the development of epithelial cells because of a large population of pluripotent ASCs in vivo.^5^, ^6^ The planarian epidermis, which is a monostratified tissue, has been identified three epidermal types: ciliated, non‐ciliated epidermis and doral–ventral boundary epidermis.^7^, ^8^, ^9^ The specialized neoblast lineage (ζneoblasts) gives rise to the epidermal lineage. In response to the amputation‐induced injury, the ζneoblast that is distributed throughout the mesenchyme produces descendants that migrate, differentiate and integrate into the epidermis to replace the damaged cells.^10^, ^11^ In addition, the epidermis is required for regeneration. The pre‐existing epidermis can stretch to cover the wound caused by amputation and initiates the process of tissue regeneration in concert with other cell types.^12^

A ζneoblast goes through several transitions defined by cellular morphology and spatial distribution to eventually form the mature epidermal cell and the organs. Thus, the spatially and temporally distinct planarian epidermis consists of a remarkable diversity of differentiated cell types.^5^ The single‐cell RNA sequencing showed that gene expression at each stage of epidermal development was different. According to the reports, the gene *prog‐1* and *agat‐1* were expressed in early and late epidermal progenitors, respectively. These abundant *prog‐1* ^+^ and *AGAT‐1* ^+^ cell populations were identified as two distinct populations of epidermal progeny cells and have been widely used to analyse neoblast differentiation.^13^, ^14^ As the assays for evaluating planarian epidermal integrity and function were well established, a number of genes controlling epidermal development were identified. There is increasing evidence suggesting that epidermal development is a dramatic and highly regulated process in which the cytoskeleton plays differing yet essential roles.^15^, ^16^ The role of the cytoskeleton in planarians is not well known.

Myosins, the main elements of the cytoskeleton are molecular motors that can generate force and movement through their interaction with actin and facilitates cytoskeletal organization.^17^ Myosin regulatory light chain (*MRLC*) is a component of the myosin and is important for the appropriate physiological functioning of myosins.^18^, ^19^, ^20^ The myosin activity leading to the contraction is stimulated by the phosphorylation of *MRLC* in distinct sites.^21^ The loss of *MRLC* phosphorylation in mice leads to defects in muscle construction and result in severe gut dysmotility.^22^ In Drosophila, the ectopic expression of phosphorylated *MRLC* causes the failure of edge cell elongation.^23^ The depletion of *MRLC* in zebrafish contributes to the changes in cardiac contractility.^24^ Additionally, *MRLC* is involved in a wide range of cellular process by controlling the formation of myosin filament and actin assemble, including cell migration, cell adhesion and cytokinesis.^25^, ^26^ However, the specific functions of *MRLC* during epidermal development are not well understood.

Here, we report the characterization of the *MRLC* homologue in planarians. Whole‐mount in situ hybridizations revealed that it is mainly expressed in wound region. Functional analysis by RNA interference (RNAi) revealed an essential function for *MRLC* in planarian tissue homeostasis and regeneration. We confirmed the expression of *MRLC* in late epidermal progenitors. RNAi knockdown of *MRLC* led to the collapse of the epidermis cell lineage, as indicated by the abnormal expression of several epidermal expressed genes. In addition, *MRLC* expression was observed in *cdc42* ^+^ cells. We found that simultaneous *MRLC* and *cdc42* RNAi dramatically increased cell death and enhanced the defects in epidermis. These results suggest that *MRLC* participates in maintaining the epidermal lineage, and functions as the regulator to control apoptotic cell death with *cdc42*.

## 2 MATERIALS AND METHODS

### 2.1 Species and culture conditions

All experiments were performed with the clonal strain of the planarian *Dugesia japonica*. Animals were kept at 20°C in autoclaved stream water as previously described.^27^ Planarians 4–6 mm in length were starved for at least 1 week prior to experiments. All animal experiments were performed according to the guidelines evaluated and approved by the ethics committee of Zhengzhou University.

### 2.2 Gene cloning and RNAi experiments

Genes in this study were cloned from a Dugesia japonica cDNA library. Primer sequences are provided in Table S1. Double‐stranded RNAs (dsRNAs) were synthesized as previously described.^28^, ^29^ In brief, dsRNA was obtained by in vitro transcription using T7 polymerase. DsRNA was administered to animals using microinjection (Drummond Scientific) twice a day for 4 days. Control animals were injected with diethypyrocarbonate‐treated H_2_O. For regeneration studies, animals were cut transversely into three pieces before and after the pharynx at 24 h after the last feed and allowed to regenerate.

### 2.3 In situ hybridization

RNA probes were in vitro synthesized using T7 polymerase (Roche) and digoxigenin (DIG)‐modified ribonucleotides (Roche). Whole‐mount ISH (WISH) and fluorescent ISH (FISH) were performed as previously described.^30^, ^31^ Animals were treated with 5% N‐acetyl‐cysteine (NAC) in PBS for 5 min at room temperature and fixed in 4% formaldehyde. Animals were bleached in 6% hydrogen peroxide overnight followed by rehydration in methanol at −20°C. Then these samples were placed in Proteinase K (20 mg ml‐1 in PBS containing 0.3% Triton X‐100) for 10 min, and fixed before incubation in pre‐hype for 2 h at 56°C and hybridized with a DIG‐labelled probe at 56°C for 16–17 h. After proper washing and blocking in 5% Western Blocking Reagent, an anti‐DIG antibody was used in a 1:1000 dilution. Digoxigenin‐ or dinitrophenol‐labelled riboprobes were detected with anti‐digoxigenin‐horseradish peroxidase (HRP) and anti‐dinitrophenol. Finally, colourimetric (NBT/BCIP) was performed to visualize the signal. In FISH staining, the signals were developed with tyramide amplification. For the regeneration experiments, the animals were cut transversely into 3 pieces including the head, trunk (including the pharynx) and tail, and the fragments were treated at 1, 3 and 7 d after amputation.

### 2.4 BrdU labelling

Animals were treated with 0.0625% N‐acetyl‐cysteine dissolved 1× Montjuic water for 30 s, rinsed animals three times with 1× Montjuic water and then incubated in 20 mg/mL BrdU in 3% dimethylsulfoxide/1× Montjuic for 1 h. Animals were killed and bleached overnight in 6% hydrogen peroxide in methanol. After fluorescence in situ hybridization development, specimens were performed in 2 N hydrogen chloride at room temperature (RT) for 45 min. Then, samples were washed with PBS and blocked in PBSTB for 4–6 h at RT. Animals were incubated overnight in rat anti‐Brdu antibody (1:1000), rinsed in PBSTB over 2 h with gentle agitation and incubated overnight in goat anti‐rat‐HRP antibody (1:1000) and tyramide amplification.

### 2.5 Whole‐mount immunostaining

Immunostaining was carried out as previously reported.^32^, ^33^ The planarians 1–5 mm in length used for experience were killed with 5% NAC in PBS (phosphate buffered saline) for 5 min at room temperature, washed three times with PBST (phosphate buffered saline containing 0.1% TritonX‐100) at RT and fixed in PBST containing 4% paraformaldehyde for 2–4 h at 4°C. Then, worms were placed in 100% methanol for 1 h at −20°C and treated with 1% SDS in PBST for 10 min at RT. After rinsed in PBST, the samples were blocked with 10% goat serum in PBST for 2 h at 4°C or RT. They were then incubated with primary anti‐synapsin (1:100; Developmental Studies Hybridoma Bank) or anti‐H3P antibodies (1:250; Millipore, 05‐817R) overnight at 4°C. The specimens were washed four times with PBST for 30 min per wash before adding the secondary antibody goat anti‐mouse Alexa Fluor488 (1:500) and goat anti‐rabbit Alexa Fluor568 (1:500). Stained planarians were visualized using a fluorescence microscope (Olympus, Tokyo, Japan).

### 2.6 Whole‐mount TUNEL

The planarians were fixed and stained for TUNEL as described previously.^34^ In brief, animals were killed with 10% N‐acetyl cysteine (diluted in PBS) for 5 min at room temperature, and fixation in 4% formaldehyde for 20 min. Animals were then permeabilized in 1% SDS (diluted in PBS) for 20 min at RT with rocking and bleached overnight at RT, without rocking, in 6% H_2_O_2_ (diluted in PBST). After rinsed in PBST, the bleached animals can store at 4°C in PBST. For staining, animals were washed in PBS and incubated in terminal transferase enzyme (Chemicon, Cat. No. 90418) diluted in reaction buffer (Chemicon, Cat. No. 90417) for 4 h at 37°C. Enzyme‐treated worms were stopped by washing in stop/wash buffer (90,419; Millipore) and washed three times with PBSTB (PBST with 0.25% bovine serum albumin) for 5 min per wash. These worms were then incubated in anti–digoxigenin‐rhodamine (90,429; Millipore) diluted in blocking solution (90,425; Millipore) for 4 h at RT. At room temperature, Stained animals were rinsed three times briefly in PBSTB for 10 min each on a platform shaker and mounted under cover slips on glass slides. The signals were visualized using a fluorescence microscope.

### 2.7 Quantitative real‐time polymerase chain reaction (PCR)

Total RNA was extracted from a pool of three RNAi‐treated planarians using TRIzol (TaKaRa) and cDNAs were synthesized with oligo‐dT primers and reverse transcriptase (TaKaRa). qRT‐PCR experiments were performed using SYBR green qPCR Master Mix (Vazyme). Experiments were performed on three biological replicates. The data were normalized based on the expression of the internal control elongation factor 2 (EF‐2).

### 2.8 Statistical analysis

Data were compared between the different test groups using Student's *t*‐test by the GraphPad Prism 5.0 software. All the experiments were repeated at least three times independently and the differences between groups were considered significant if *p < 0.05 and **p < 0.01. NS, not significant.

## 3 RESULTS

### 3.1 Spatiotemporal expression pattern of *MRLC* in intact and regenerating planarians

**FIGURE 1:**
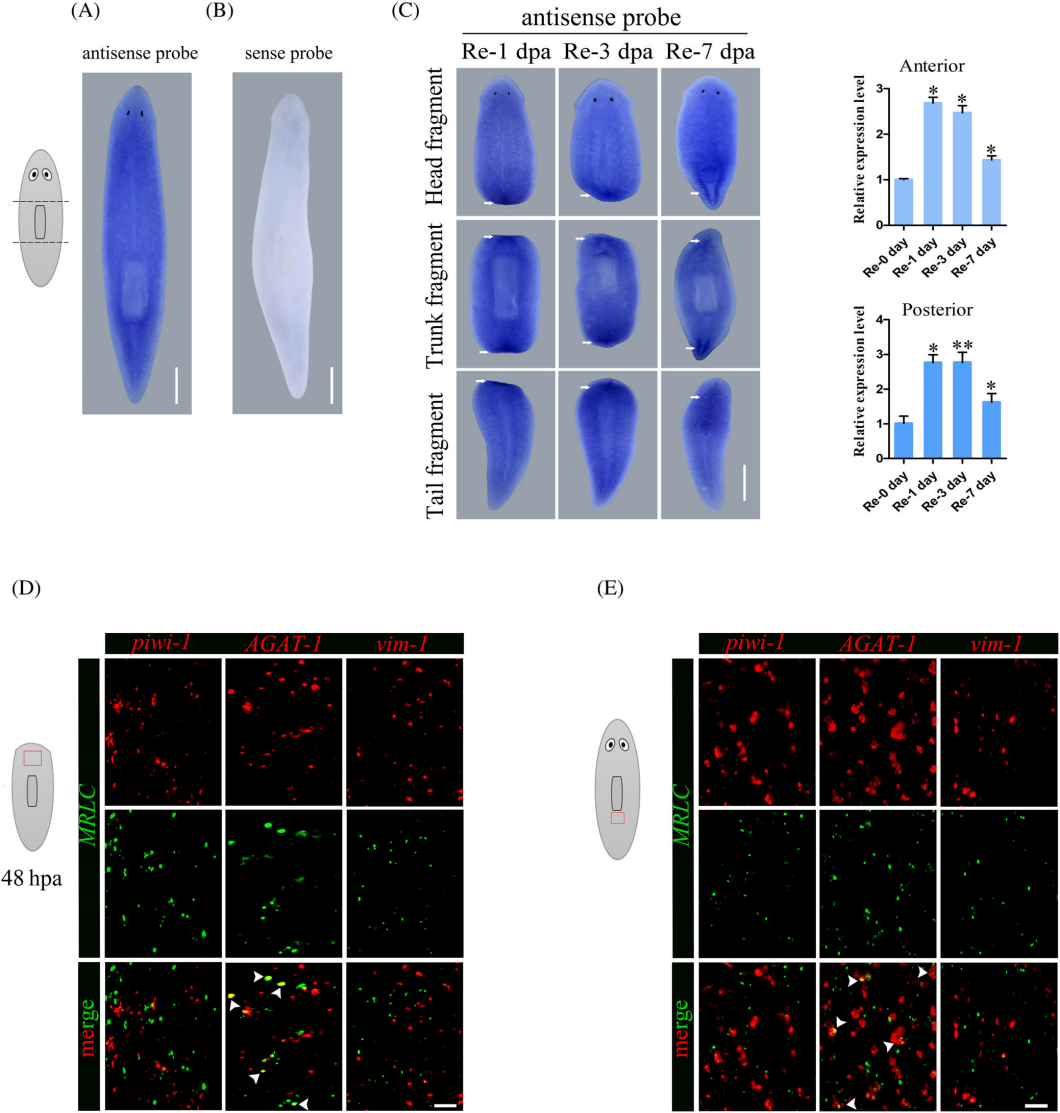
Expression pattern of *MRLC* during planarian regeneration. (A) WISH with antisense *MRLC* probe in uninjured animals (*n* ≥ 10). The ubiquitous expression patterns were observed. (B) WISH with sense *MRLC* probe in intact planarian showed the absence of hybridization signal. (C) Temporal pattern of *MRLC* expression during regeneration. Wild‐type animals were cut transversely into three pieces before and after the pharynx, and *MRLC* expression was analysed using Whole‐mount in situ hybridization. Strong expression of *MRLC* is mainly in wound region at 1, 3 and 7 days following wounding. Quantification of *MRLC* expression levels in the anterior and posterior end in regenerants from head and tail fragments were measured through qPCR. The white arrows point to higher expression in the wound region. Each stain had ≥10 worms assayed. qRT‐PCR data obtained from biologically and technically triplicated. *p < 0.05; **p < 0.01. (D–E) Double fluorescent in situ hybridization of *MRLC* combined with markers of different stages of epidermal development in regenerating and normal animals. *MRLC* expression in late epidermal progenitors. Double‐positive cells are denoted with white arrows. The red box in the cartoon depicts the region imaged. Each stain had ≥10 worms assayed. Scale bars: 200 μm in (A–C). 50 μm in (D–E).

We identified the *MRLC* homologue in the D. japonica transcriptome (OM234687, Figure S1). To investigate the roles of *MRLC*, we first analysed the spatiotemporal expression pattern of *MRLC* in intact and regenerating planarians using whole‐mount in situ hybridization. The regenerating fragments were obtained using transverse dissection into three pieces before and after the pharynx, and fixed at 1, 3 and 7 day post‐amputation. In intact worms, we observed the expression of *MRLC* was ubiquitous with no localization to specific organs (Figure 1A). In addition, the absence of a hybridization signal with sense *MRLC* further validated the specificity of *MRLC* expression (Figure 1B). During regeneration, the transverse sections of animals stained for *MRLC* showed strong expression in wound region at day 1 after amputation (Figure 1C). Over a 3‐day period, the high expression levels of *MRLC* in the new head and tail region were continuous (Figure 1C). When the worms regenerated into complete individuals 7 days after amputation, the expression of *MRLC* was mainly distributed in the anterior and posterior to the newly formed pharynx and the pharynx‐forming region (Figure 1C). The quantification of *MRLC* level in the anterior and posterior end in regenerants from head and tail fragments showed that the expression of *MRLC* was increased (*n* ≥ 10 worms; Figure 1C). Additionally, the single‐cell transcriptomic approaches combined with computational algorithms have been used to generate an atlas of cell type transcriptome, which indicated the expression of *MRLC* in the epidermis.^35^ To further analyse the cell types expressing *MRLC*, we detected the expression of *MRLC* by colocalization studies with markers of different stages of epidermal development in regenerating and intact animals. The results showed the expression of *MRLC* in late epidermal progenitors, which agrees with published single‐cell RNA‐seq databases (Figure 1D, E). These data raise the possibility that *MRLC* is a generically wound‐induced gene and may play a functional role in epidermal development.

### 3.2 Knockdown of *MRLC* causes tissue defects in homeostasis and regeneration

**FIGURE 2:**
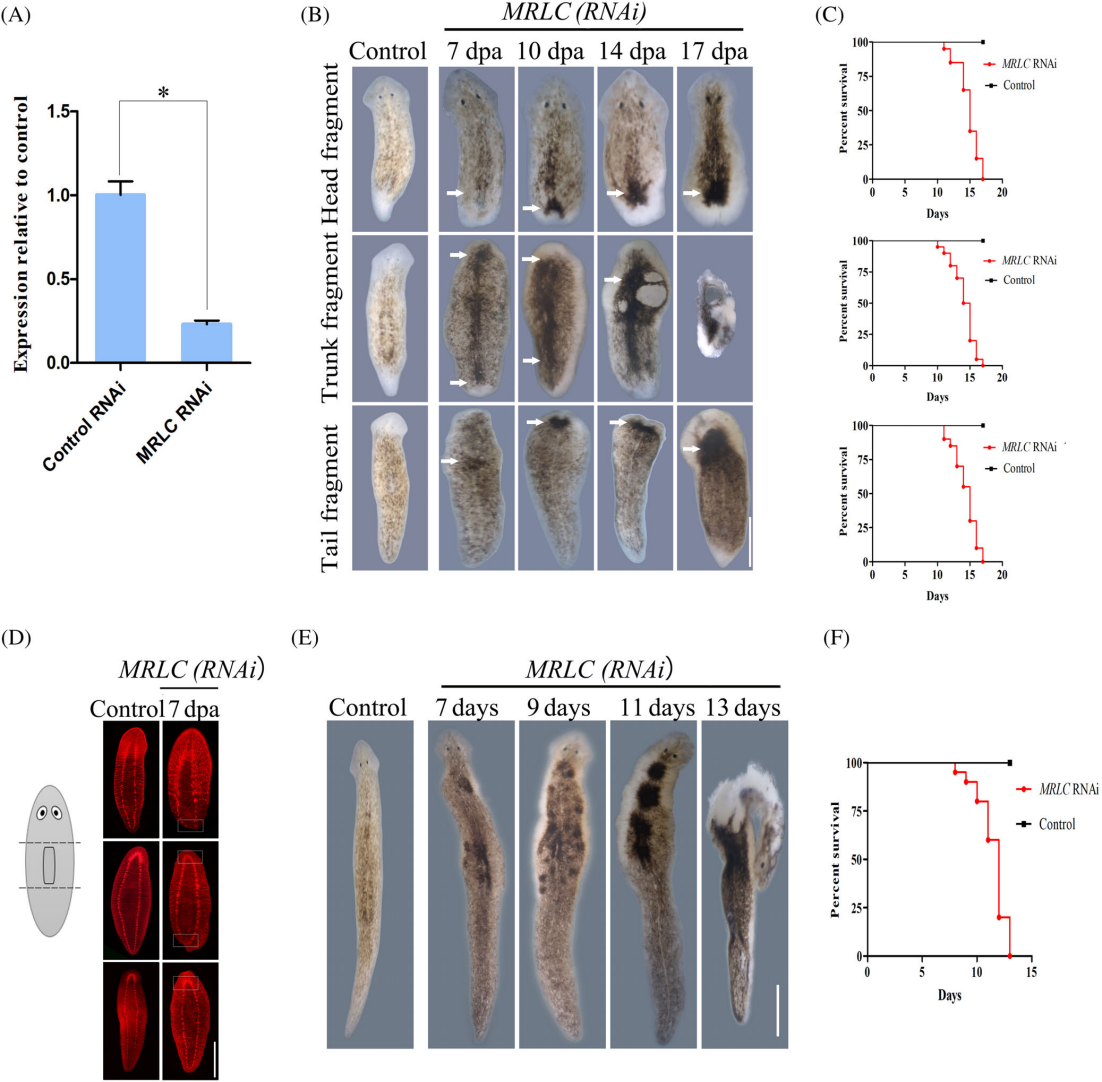
*MRLC* is required for planarian regeneration and tissue homeostasis. (A) qRT‐PCR to measure *MRLC* transcript levels after RNAi. The *MRLC* transcript levels were reduced at 2 days after the last injection. The experiments were biologically and technically triplicated. At least 30 animals were assayed. Error bars represent standard errors of the SD;*p < 0.05. (B) Regeneration phenotypes of heads, trunk and tail fragments after treatment with control dsRNA or *MRLC* dsRNA (*n* = 20). As compared with control (RNAi) worms, *MRLC* (RNAi) regeneration is severely deficient. White arrows point to the defects in epidermis. Scale bars: 200 μm. (C) Survival curves for control and *MRLC* (RNAi) animals during regeneration (*n* = 20). (D) Immunostaining with anti‐synapsin antibody in regenerating fragments 7 days after amputation (*n* = 8). White boxes indicate the defects in the central nervous system. Scale bars: 200 μm. (E, F) Intact phenotypes for control and *MRLC* RNAi animals during normal tissue turnover (*n* = 20). The uninjured experimental worms generated severe abnormalities, characterized by darkening of epidermis. The worms completely lost their heads by day 11 and eventually lyse. Survival curves for control and *MRLC* (RNAi) animals are on the right. Scale bars: 300 μm.

To get a better understanding of the role of *MRLC* in planarian, RNAi knockdown experiments were performed in intact and regenerating animals. Injecting the planarians with double‐stranded RNA (dsRNA) was performed. The *MRLC* transcript levels were examined by qPCR to verify the down‐regulation efficiency. We found a fivefold reduction in *MRLC* transcript levels 2 days after the last injection (Figure 2A). We chose the time points after dsRNA injections at which the worms were transversely amputated into three pieces, including head, trunk and tail fragments. At 7 days post‐amputation, control animals regenerated the missing body parts completely (Figure 2B). In contrast, the fragments treated with *MRLC* dsRNA displayed aberrant regeneration after amputation. We observed a range of deformities: loss of epidermal integrity, the darker epidermis and complete loss of the anterior or posterior (100% penetrance; *n* = 20; Figure 2B). *MRLC* RNAi worms exhibited the curling phenotype, which proceeded until the worms were completely lysed (Figure 2B). Additionally, the defects in the regeneration of *MRLC* RNAi planarians were characterized by examining the expression pattern of anatomical marker. The expression of synapsin, a central nervous system (CNS) marker, showed a defective cephalic brain in trunk and tail fragments, although ventral nerve cords (VNCs) did regenerate (Figure 2D). The ventral nerve cords in the head fragments were truncated (Figure 2D), likely due to the failure of posterior regeneration or tissue degradation. These data indicate that *MRLC* plays an essential role in the proper regeneration in planarians.

We next analysed the function of *MRLC* in tissue homeostasis. Following *MRLC* RNAi, we found that uninjured worms generated severe abnormalities during homeostasis, characterized by darkening of epidermis (20/20 animals; Figure 2E). The experimental group undergoes the formation of lesion and deterioration, complete loss of their heads and eventual lysis (Figure 2E). These data further suggest that *MRLC* is required for tissue homeostasis. In agreement with this, the survival analysis indicated that *MRLC* RNAi animals showed a significant reduction in survival during regeneration and homeostasis (Figure 2C, F). It has been proved that *MRLC* regulated morphogenesis by controlling myosin activity.^36^ Studies have shown that the defects of lesions and/or ventral curling are often correlated with impaired epidermal differentiation.^37^ Combined with these gross morphological defects, we proposed the possibility that the knockdown of *MRLC* caused the defects of epidermal development. It may influence the epidermal cell lineage and cause an imbalance between stem cell progeny and proliferation. Thus, we next further assayed the changes in epidermal lineage markers to identify the effect of *MRLC* on cell division and the maintenance of epidermal lineage.

### 3.3 The *MRLC* (RNAi) results in hyper‐proliferation and misexpression of epidermal progeny markers

**FIGURE 3:**
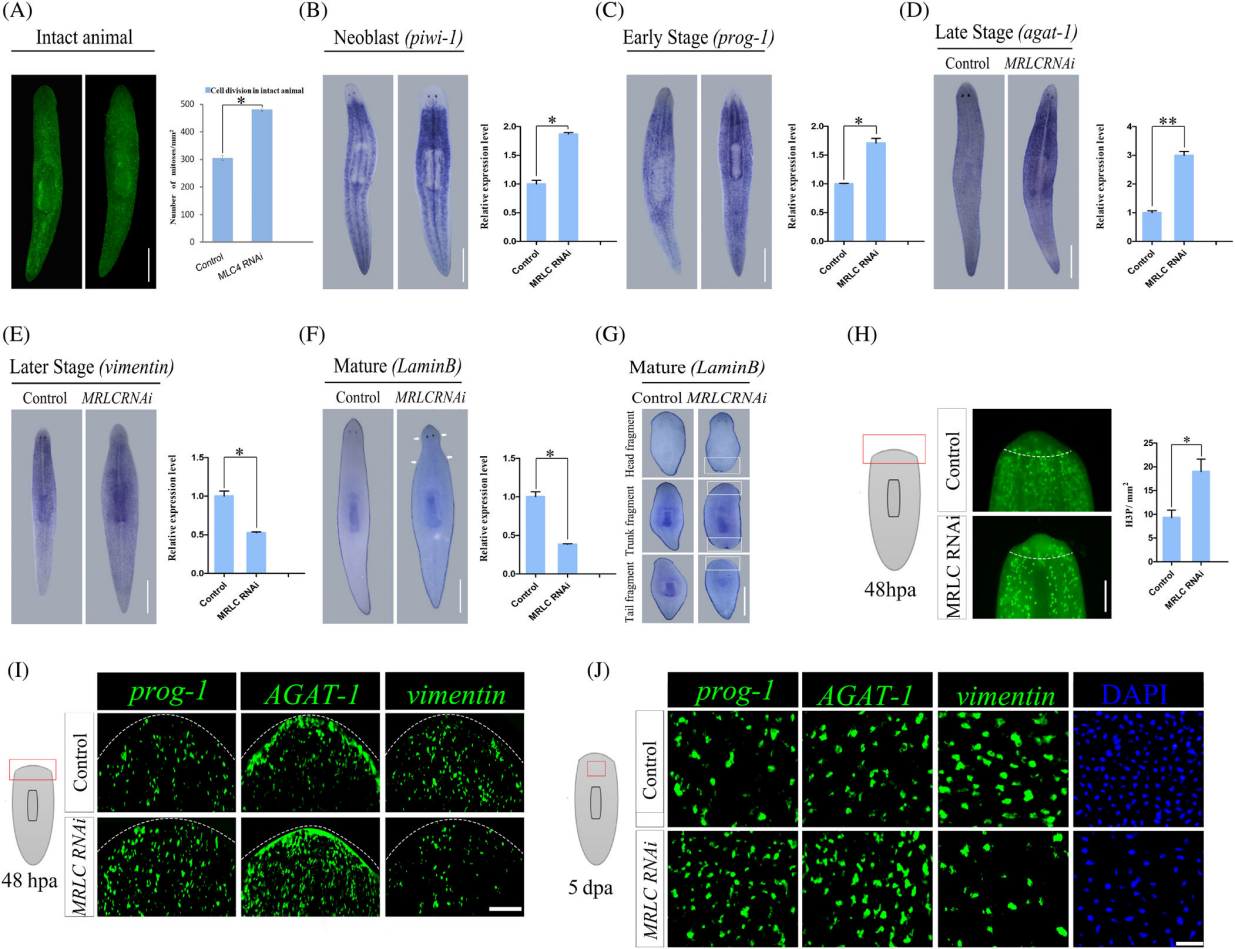
*MRLC* is required for cell proliferation and the proper differentiation of epidermal progeny cell. (A) Animals were stained at 7 days after last injecting, using the marker H3ser10p, which marks dividing cells during the G2/M transition of the cell cycle. Quantification of H3P‐positive cells in control and *MRLC* (RNAi) intact animals on the right (*n* = 8). Scale bars: 200 μm. (B–D) Examination of stem cell and epidermal progenitor population at 7 days post‐RNAi in intact worms. Whole‐mount in situ hybridization (WISH) of lineage markers for stem cells *piwi‐1*, early progeny *prog‐1* and late progeny *AGAT‐1* (*n* ≥ 10). Scale bars: 200 μm. (E, F) Analysis of later progeny marker *vimentin* and the mature epidermal cell‐specific marker *LaminB* by WISH during epidermal differentiation (*n* ≥ 10). (B–F) Quantification of the level of expression of *piwi‐1*, *prog‐1*, *AGAT‐1*, *vimentin* and *LaminB* by qRT‐PCR. *n* = 30 worms with biological triplicates. *p < 0.05, **p < 0.01; Scale bars: 200 μm. The error bars indicate SD. (G) Examination of the epidermal marker *LaminB* by WISH in regenerating head, trunk and tail fragments in RNAi worms (*n* ≥ 10). Animals were transversely dissected into three pieces before and after the pharynx at last injecting and fixed at 7 days post‐amputation. At least eight biological replicates were used. Scale bars: 200 μm. (G‐F) The white arrows and boxes indicate the loss of the mature epidermal cell population. (H) whole‐mount immunofluorescence for phosphorylated histone H3 (H3ser10p) in *MRLC* RNAi regenerating worms 48 h post‐amputation (*n* = 8). The number of PhosphoH3+ cells in the regenerating blastema of *MRLC* RNAi animals was increased compared with control animals. The red box in cartoon depicts the region imaged. The White dotted line indicates the wound boundary. Scale bars: 200 μm. (I) Analysis of early progeny, late progeny and later progeny markers by fluorescent in situ hybridization in the regenerating blastemas of RNAi worms at 48 h (*n* ≥ 8). White dashed lines outline the animals. Scale bars: 200 μm. (J) Epidermal populations are assayed by FISH for *prog‐1*, *AGAT‐1*, *vimentin* and DAPI at 5dpa (*n* ≥ 8). Scale bars: 50 μm.

Previous studies have reported that the defects in regeneration and homeostasis in planarian are often a consequence of the aberrant stem cell dynamics, including abnormal cell proliferation.^38^, ^39^ To determine whether the defects in *MRLC* RNAi worms were attributed to neoblast division, whole‐mount immunofluorescence was performed. The time point was chosen on day 7 before lyse. Immunostaining against phosphorylated histone 3 (H3P), a dividing cells maker beginning at the G2/M transition of the cell cycle,^39^ showed that neoblast proliferation in intact animals was significantly increased compared to controls for 7 days post‐RNAi (Figure 3A). This indicates that *MRLC* is required for proper cell proliferation. Two hypotheses that can explain hyper‐proliferation are the perturbation of stem cells or their progeny. First, hyper‐proliferation might be occurred by failure of stem cell differentiation into progeny. Second, the failure of progeny to differentiate or survive might signal neoblast to excessive proliferation to compensate for the lack of progeny.

To resolve whether the stem cell hyper‐proliferation was the failure of differentiation and determine which cell types were affected, the expression patterns of genes marked by epidermal cells were assayed in the intact *MRLC* RNAi animals. The planarian *piwi‐1* is expressed in dividing cells, marking neoblasts.^40^, ^41^ WISH analysis revealed that *MRLC* RNAi animals exhibited the dramatic increase in stem cells compared with controls at day 7 (Figure 3B). In the model of epidermal differentiation, the post‐mitotic descendants emerge from a specialized subclass of neoblast. The epidermal lineage transition through three characterized differentiation stages—early progeny, late progeny and later progeny—before integrating into epidermal. The *prog‐1* ^+^ (early progeny) and *AGAT‐1* ^+^ (late progeny) postmitotic cell populations have been identified which are part of an epidermal lineage.^5^ Thus, we assessed the effect of *MRLC* on epidermal lineage differentiation and found that the two epidermal lineages of early progenitor marker *prog‐1* and late progenitor marker *agat‐1* showed a significant increase in staining, attributable to increased cell numbers (Figure 3C, D). These experiments suggest that the increase in stem cell population in *MRLC* RNAi animals is not at the expense of the early and late progeny.

Previous works have identified that *vimentin* and *LaminB*, which mark post‐mitotic cell, is downstream of the *prog‐1* and *agat‐1* epidermal lineage.^10^ The fact that the increase in stem cell population was not the failure of stem cell differentiation into progeny. Therefore, it was possible that *MRLC* is required for the later stage of epidermal differentiation. To test this hypothesis, we examined the expression of *vimentin* which marked the later progeny during epidermal differentiation. In *MRLC* RNAi animals, the downregulation of *vimentin* expression was detected (Figure 3E). Consistent with this, the assay of the mature epidermal cell population in *MRLC* RNAi animals by the expression of the mature epidermal cell‐specific marker *LaminB* showed a dramatical decrease in staining at the edge of the boundary (Figure 3F). Similar results were obtained by analysing the expression levels of the same markers by real‐time RT‐PCR (Figure 3B–F). We also investigated the expression of *LaminB* at 7 days post amputation and found the observable change in *MRLC* RNAi animals, which showed that *MRLC* may be crucial for epidermal development during regeneration (Figure 3G).

Amputation triggers neoblast proliferation in two distinct phases. The second proliferative phase which occurred at 48 h post amputation is crucial for blastema formation.^42^ We assayed the mitotic cells at 48 hpa in *MRLC* RNAi animals. The number of the H3P^+^cells was increased in the regenerating blastema (Figure 3H). To further test for possible effects of *MRLC* inhibition on the rate of epidermal differentiation, we examined the spatial distribution of *prog‐1*, *AGAT‐1* and *vimentin* epidermal markers by FISH and found that the early and late progeny cells were strongly increased and epidermal later progeny cells were lost (Figure 3I). Quantification of *prog‐1* ^+^, *AGAT‐1* ^+^and *vimentin* ^+^ cells was consistent with FISH analyses (Figure S2A). Moreover, the defects in epidermal development can also be observed at 5 days after amputation (Figures 3J and S2B). Given the severity of phenotypic defects, we investigated the epidermal nuclear density to determine the problems with epidermal density and found that *MRLC* RNAi animals exhibited a dramatically reduced number of epidermal cells (Figures 3J and S2B). These results indicate that *MRLC* is required for maintaining the epidermal lineage development in both regeneration and homeostasis. The hyper‐proliferation in *MRLC* RNAi animals might be induced by promoting cell division to compensate for the decreased epidermal cells.

**FIGURE 4:**
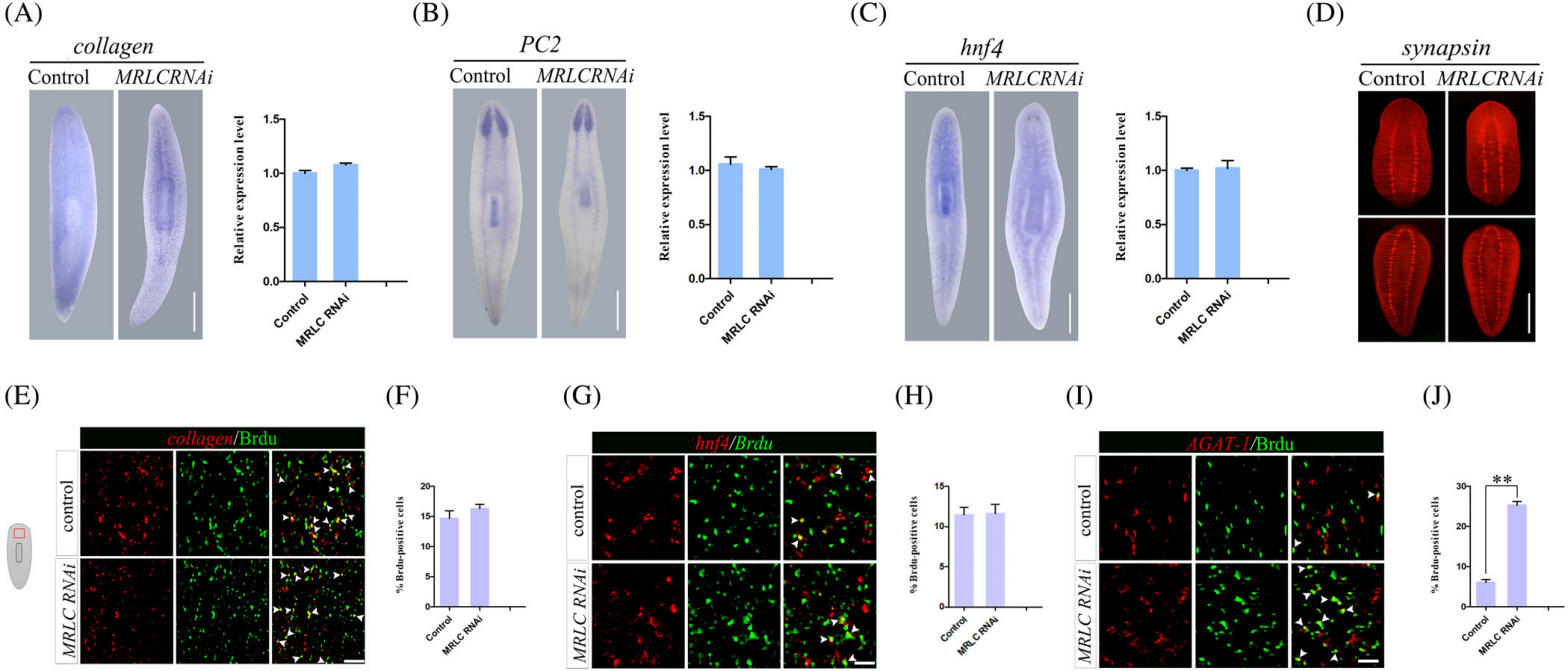
*MRLC* (RNAi) animals display a marked increase in epidermal cells without affecting the regeneration of muscle, nervous system and gut. (A–C) Analysis of muscle marker and anatomy markers by WISH and qPCR in control and *MRLC* RNAi intact animals fixed at day 7 after the last injecting (*n* ≥ 10). Scale bars: 200 μm. (D) Immunostaining with anti‐synapsin, which visualize the central nervous in regenerating head and tail fragments 3 days after amputation (*n* ≥ 8). Scale bars: 200 μm. (E–J) FISH for BrdU and different cell (*collagen*, *hnf4* and *AGAT‐1*) in control and *MRLC* knockdown worms. The worms were soaked with Brdu at 5 days after amputations, fixed animals after 24 h. The histogram depicts the percentage of colocalized cell in control and *MRLC* RNAi worms (F, H, J) (*n* = 8). The red box in the left cartoon depicts the region imaged. Double‐positive cells are marked with white arrowheads. **p < 0.01. Scale bars: 50 μm.

In order to identify whether other tissues or organs were also affected, the morphological phenotypes upon knockdown were investigated 7 days after the final injecting by whole mount in situ hybridization with the nervous system marker *PC2*,^43^ the gut marker *hnf4* ^44^ and muscle marker *collagen*.^45^ *MRLC* RNAi animals did not observe any significant differences in muscle and CNS anatomy and gut morphology compared to controls (Figure 4A–C). Because of the formation of neural circuits at 2–3 days of regeneration,^46^ we examined the nervous system in *MRLC* RNAi animals at 3 days after amputation and found *MRLC* RNAi animals showed normal expression of synapsin (Figure 4D). Then, we performed bromodeoxyuridine (BrdU) labelling on regenerative worms. The neoblasts are the only proliferating cells, so BrdU initially marks these cells and subsequently their newly born post‐mitotic progeny. We soaked animals with BrdU at 5 days after amputations, fixed animals after 24 h and examined the distributions of differentiating progeny cells by FISH and Brdu incorporation. In accordance with WISH, *MRLC* RNAi animals had *MRLC* approximately normal numbers of BrdU^+^ *hnf4* ^+^ and BrdU^+^ *collagen* ^+^ compared to controls during regeneration (Figure 4E–H). By contrast, the ratio of BrdU^+^ *AGAT‐1* ^+^ cells to the total amount of BrdU^+^ cells was remarkably increased in *MRLC* RNAi animals (Figure 4I, J). Together, our results demonstrate that *MRLC* can be directly involved in the epidermal development and *MRLC* knockdown causes defects in epidermal development and further leads to a range of deformities and worm lysis.

### 3.4 *MRLC* suppresses excessive apoptotic cell death for proper body remodellings after amputation

**FIGURE 5:**
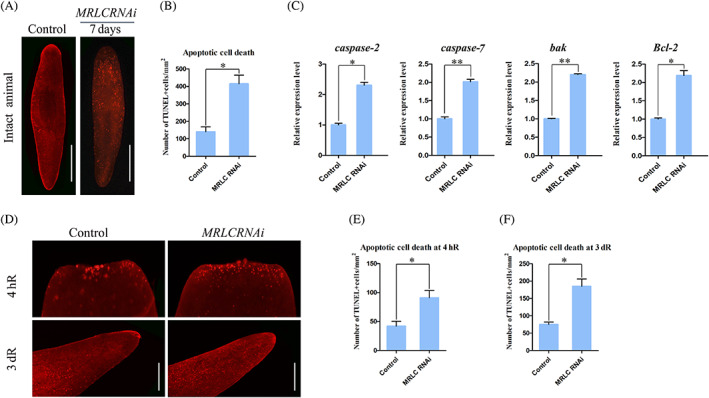
*MRLC* (RNAi) alters apoptotic cell death pattern in planarian regeneration and tissue homeostasis. (A) TUNEL staining measuring apoptosis in control (RNAi) and *MRLC* (RNAi) intact animals at day 7 post‐RNAi (*n* ≥ 8). (B) Quantification of TUNEL‐positive cells over the surface area in (A). (C) qRT‐PCR analysis quantifying *caspase‐2*, *caspase‐7*, *bak* and *Bcl‐2* expression at 2 days after the last injecting. *n* = 30 worms with biological triplicate. *p < 0.05, **p < 0.01; The error bars indicate SD. (D) Who‐mount TUNEL staining showing apoptotic cells in the wound region (4 hR) and in pre‐existing regions (3dR) post‐amputation in the control (RNAi) and *MRLC* (RNAi) worms. (E, F) Quantification of TUNEL‐positive cells over the surface area in (D) showing excessive apoptotic cell death in the experimental group. At least 10 biological replicates were used per time point. Error bars represent standard errors of the mean; *p < 0.05; Scale bars: 300 μm.

The cellular and molecular mechanisms that regulate and coordinate stem cell proliferation, differentiation and cell death are crucial for the regeneration of missing tissues and homeostatic tissue turnover. To further resolve whether hyper‐proliferation was specifically a failure of progeny to survive, terminal deoxynucleotidyl transferase deoxyuridine triphosphate nick end labelling (TUNEL)^47^ were performed. *MRLC* (RNAi) planarians exhibited a remarkable increase in cell death (Figure 5A). Correspondingly, the TUNEL‐positive cells we quantified corroborated the significant increase during *MRLC* RNAi knockdown (Figure 5B). We quantified the expression levels of *caspase‐2*,^48^ *caspase‐7*,^49^ the pro‐apoptotic gene *bak* ^50^ and antiapoptotic gene *Bcl‐2*,^51^ which are reported to be involved in apoptosis. qPCR quantification showed their upregulation in *MRLC* RNAi animals (*n* = 30 animals; Figure 5C). These results suggest *MRLC* plays a crucial role in controlling apoptotic cell death. Accordingly, we proposed that excessive apoptotic cell death in intact *MRLC* (RNAi) animals may result in the phenotypes of tissue degradation and decreased epidermal cells. Overall, *MRLC* is essential to cellular survival and tissue maintenance.

Cell death is an important component of remodelling during regeneration. Cell death releases regeneration signals that induce cellular responses and stimulate the proliferation of stem cells and progenitor cells when injured.^52^, ^53^ Recent studies indicate that during planarian regeneration, two distinct phases of cell death are observed. The first peak occurred around 4 h post‐amputation in the wound region, which is specific to wound healing, whereas the second peak, recorded 3 days after amputation, that spread throughout the organism is specific to regeneration.^47^, ^54^ Using the TUNEL assay, we analysed apoptotic cell death at 4 h after anterior amputation, and found that *MRLC* RNAi showed a significant increase in TUNEL‐positive cells compared to control (Figure 5D). There was also an apparent increase in pre‐existing tissue 3 days after amputation (Figure 5D). Consistent with these, we quantified TUNEL‐positive cells and found that cell death was increased in *MRLC* RNAi animals (Figure 5E, F). The local and systemic cell death responses are stem cell‐independent and occur predominantly in differentiated cells. Thus, we reasoned that the excessive apoptotic cell death in *MRLC* RNAi is probably responsible for the reduced epidermal cell and complete failure in regeneration. Taken together, these data indicate *MRLC* is essential to controlling wound‐induced apoptotic and further indicate that *MRLC* is required for tissue homeostasis and proper tissue remodelling.

### 3.5 *MRLC* and *cdc42* are required for epidermal lineage during homeostasis and regeneration

**FIGURE 6:**
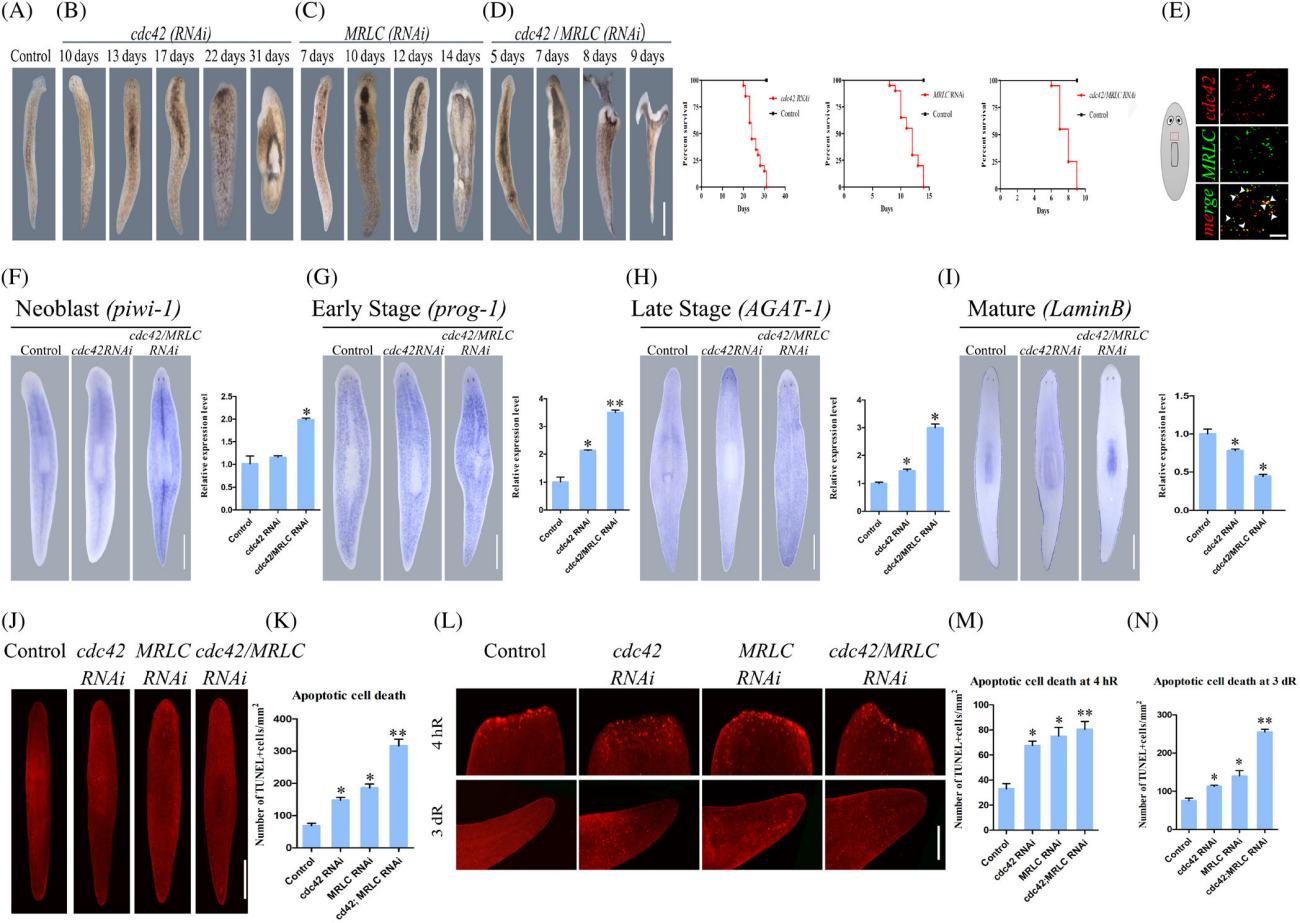
*MRLC* and *cdc42* synergize to control epidermal development. (A–D) Single and double‐RNAi as indicated to examine interactions between *MRLC* and *cdc42*. The total concentrations of dsRNA were normalized with the control dsRNA. Double inhibition of *MRLC* and *cdc42* led to pronounced phenotypic defects and accelerated worm lysis. Survival curves for control and *cdc42* (RNAi), *MRLC* (RNAi) and *cdc42*/*MRLC* (RNAi) animals (*n* = 20 at each RNAi condition). Scale bars: 300 μm. (E) Double FISH, *cdc42* (red) and *MRLC* (green) in wild animals (*n* = 10). Red boxes represent the region imaged. White arrowheads highlight double‐positive cells. Scale bars: 50 μm. (F–I) Representative WISH images of RNAi animals with a corresponding qRT‐PCR for neoblast (*piwi‐1*), early progeny (*prog‐1*), late progeny (*AGAT‐1*) and mature epidermal cell (*LaminB*). Each stain had ≥10 worms assayed. qRT‐PCR data obtained from biologically and technically triplicated. *p < 0.05, **p < 0.01; Scale bars: 200 μm. The error bars indicate SD. (J) Cell death in uninjured animals was assayed by TUNEL staining at day 5 post‐RNAi (*n* = 10). Animals were treated with control, *MRLC* and *cdc42* dsRNA by injection. Scale bars: 300 μm. (K) Quantification of TUNEL‐positive cells over the surface area in (J). *p < 0.05, **p < 0.01. (L) Who‐mount TUNEL staining showing apoptotic cells in the wound region (4 hR) and in pre‐existing regions (3dR) post‐amputation in the single or double genes inhibition worms (*n* = 10). simultaneous *MRLC* and *cdc42* RNAi dramatically increased the number of TUNEL‐positive cells. Scale bars: 300 μm. (M, N) Quantification of TUNEL‐positive cells over the surface area in (L). At least eight biological replicates were used per time point. Error bars represent standard errors of the mean; Each asterisk or double asterisk indicates statistically significant differences between the experiment group and control group. *p < 0.05; **p < 0.01; Scale bars: 300 μm.

*Cdc42*, a number of the Rho GTPase, was reported that it is involved in the regulation of morphogenesis as an important regulator of cytoskeleton organization.^55^ In addition, *MRLC* and *cdc42* play important roles in myosin activity.^56^, ^57^ *Cdc42* has also been proven to regulate the epidermal development in planarians in our previous study.^58^ To identify the possible relationship between *MRLC* and *cdc42* in planarians, we conducted double gene knockdown by coinjection of dsRNA (Figure S3). Compared with single‐gene RNAi animals, the defects in tissue homeostasis were most pronounced following the simultaneous disruption of the function of *cdc42* and *MRLC*. Simultaneous inhibition of *cdc42* and *MRLC* caused the morphological defects at 5 days after RNAi, which was earlier as compared to *cdc42* or *MRLC* inhibition alone (Figure 6A–D). Double RNAi enhanced the phenotype of epidermis and accelerated worm lysis (Figure 6D). We subsequently performed double FISH to determine whether *MRLC* is co‐expressed with *cdc42*. Indeed, we observed that *MRLC* was expressed in the late epidermal progenitors as our previous finding and most *MRLC* ^+^ cells co‐expressed the gene *cdc42* (Figure 6E). The significant changes in the expression of epidermal lineage markers in *cdc42/MRLC* (RNAi) animals further suggest *MRLC* and *cdc42* could have synergistic effect in epidermal development (Figure 6F–I). Moreover, the evaluation of cell death with the TUNEL assay revealed that both individual and simultaneous knockdown of the genes was able to alter cell death versus controls (Figure 6J). Quantification of cells dying by apoptosis (TUNEL^+^) in animals showed simultaneous *MRLC* and *cdc42* RNAi dramatically increased the number of TUNEL‐positive cells relative to single RNAi of the genes (Figure 6K). In addition to causing a significant increase in intact animals, the knockdown of these two genes also affects apoptosis during regeneration (Figure 6L). We quantified TUNEL‐positives nuclei in the wound region and regenerating fragments and found that *cdc42*/*MRLC* (RNAi) did not observe significant differences compared to the single gene inhibitions at 4 h after injury (Figure 6M). Cell death around 3 days post‐amputation remained at similar levels among the single gene knockdown, whereas the double RNAi resulted in a noticeable increase in cell death (Figure 6N). Together these data lend further support to our point that *MRLC* acts synergistically with *cdc42*, whether directly or indirectly, to control epidermal development.

## 4 DISCUSSION

Adult stem cells (ASCs) are pluripotent cells and can self‐renew and differentiate into specific cell types to replace cells lost to normal physiological turnover or injury. Understanding the mechanisms that how ASCs elaborate specific programmes of post‐mitotic differentiation to produce functional mature cell types is of paramount importance to regeneration and organismal homeostasis. The planarian epidermis is an experimentally tractable system to study complex dynamics because the epidermal maturation has multiple transition states. In this study, we identified *MRLC* as an important regulator of the differentiation stages during epidermal development. The removal of *MRLC* by RNAi results in excessive cell death and the decreased epidermal cell population and triggers the hyper‐proliferation of neoblast and epidermal progenitors.

Previous studies have demonstrated that *MRLC* plays several important roles in the maintenance of the cytoskeleton and various cellular functions.^59^, ^60^, ^61^ In Drosophila, the gene mutants encoding *MRLC* block cell proliferation.^62^ The reduction of *MRLC* in the mammalian cell results in the failure of cytokinesis.^63^ In addition, *MRLC* is also necessary for tumour cell proliferation.^61^ In this report, we identified that *MRLC*, expressed in late differentiated epidermal cells (*AGAT‐1*), is required for cell proliferation and epidermal cell differentiation in the planarian. Strikingly, *MRLC* knockdown animals caused the defect mainly in epidermal tissue indicated by darkening of epidermis and the decreased epidermal cell population (Figure 2B, E, 3E–G). Whole‐mount immunofluorescence for cell division showed a dramatic increase in stem cells (Figure 3A). This result was also supported by the in‐situ hybridization experiments, which showed the expression of *piwi‐1* in *MRLC* (RNAi) worms was increased (Figure 3B). We observed the expression of the other epidermal progenitor markers *prog‐1* and *agat‐1* was also significantly increased after RNAi during regeneration and tissue homeostasis (Figure 3C, D, I, J). Furthermore, the Brdu‐labelling experiments showed the increased late epidermal progenitors in *MRLC* RNAi animals (Figure 4I, J). However, the gut as well as the muscle and nervous system remained unaffected (Figure 4A–H). The expression of *MRLC* was also observed in the epidermal cells, which suggests that *MRLC* might be directly involved in epidermal development. It has been reported that *MRLC* plays a critical role during wound healing.^64^, ^65^ The general early wound response during planarian regeneration depends on the polarity signal that stimulates cell proliferation and differentiation.^66^, ^67^ In *Drosophila*, *MRLC* is involved in the establishment and maintenance of polarity through di‐phosphorylation.^68^ *MRLC* is also essential for anterior–posterior polarity during *Caenorhabditis elegans* embryogenesis.^69^ Combined with the expression pattern of *MRLC* (Figure 1C), *MRLC* is crucial for regeneration and the downregulation may disrupt the regionalized transcriptional programmes activated by the polarity signal in the epidermis.

The balance of cell proliferation and cell death is important for generating a proportioned organism and maintaining the appropriate body size during injury or starvation. Previous study suggested that *MRLC* is critical for controlling cell death.^70^ We identified the central role for *MRLC* in regulating cell death during tissue homeostasis and regeneration. When *MRLC* is knocked down, marked increases in TUNEL‐positive cells were found in regeneration and tissue homeostasis (Figure 5A–F). The tightly regulation of injury‐induced cell death in time and space is essential for regeneration. The excessive apoptosis in regeneration is probably responsible for the failure of regeneration, worm lysis and the reduced epidermal cell. Our results indicate that *MRLC* is required for controlling injury‐induced apoptosis. In agreement with the essential role of apoptosis during planarian regeneration, *MRLC* also mediates apoptotic cell death during epidermal development. A tempting explanation for the expansion of neoblasts and multiple progenitor populations is that the expansion could be a response to the global decrease in epidermal cells, which is induced by excessive apoptosis.

Earlier studies have reported *MRLC* is implicated in cytoskeletal regulation together with *cdc42*.^71^ They were involved in actin filament stabilization and action‐myosin contraction.^72^ The changes in the expression of *cdc42* and *MRLC* lead to cell contractility attenuation, which contributes to tumorigenesis.^73^ Owing to the similarity of expression pattern and epidermal phenotypes in *MRLC* and *cdc42* knockdown animals, we hypothesize that *cdc42* acts synergistically with *MRLC* to regulate epidermal development and tissue remoulding. We observed the detectable expression of *cdc42* in *MRLC* ^+^ cells (Figure 6E). When both genes are knocked down by RNAi, severe phenotypic defects were observed compared with single RNAi of the genes (Figure 6A–D). Finally, a strong synergy relationship between *cdc42* and *MRLC* was confirmed through the WISH of epidermal lineage markers and TUNEL assay (Figure 6F–N). However, it is unknown whether the cooperation between *cdc42* and the *MRLC* in planarian or other species is direct or indirect. Thus, the precise biochemical interactions should be further explored in future studies.

The epidermis is located on the surfaces of organs and is well characterized for protective function. The epidermal cells provide the first response and cover the wound after amputation or injury.^74^ The planarian epidermis derives from a single specialized neoblast lineage and has distinct spatial and temporal differentiation stages.^10^, ^75^ The positive balance between cell proliferation, differentiation and cell death is critical for epidermal development. Our studies illustrate that *MRLC* as an inhibitor of apoptosis is required for the epidermal cell lineage development. We have identified the crucial role in controlling injury‐induced apoptosis during regeneration. When *MRLC* function is removed, the increased cell death in injured worms lead to the decrease of epidermal cell and induce hyper‐proliferation of the epidermal progenitors. This striking imbalance between cell death and proliferation destroys the maintenance of the epidermal lineage and eventually results in the lysis of animals. Our studies uncover the previously unreported regulatory mechanism of *MRLC* in epidermal development and further elucidate the role of the cytoskeleton in epidermal morphogenesis.

## AUTHOR CONTRIBUTIONS

Yujia Sun performed the experiments, analysed the data and wrote this manuscript; Yujia Sun and Yongding Huang and Zhitai Hao performed the experiments and analysed the data; Zhitai Hao performed statistical analyses; Qingnan Tian and Shoutao Zhang designed the experiments, interpreted the data and revised the manuscript for intellectual content. All authors have read and agreed to the published version of the manuscript.

## FUNDING INFORMATION

This research was funded by the National Natural Science Foundation of China (31970419), Bingtuan Science and Technology Project (2019AB034) and Scientific and technological innovation talents in Colleges of Henan (21HASTIT034).

## CONFLICT OF INTEREST STATEMENT

The authors declare no competing financial interests.

## Supporting information


**Data S1.** Supporting Information.

## Data Availability

The data that support the findings of this study are available from the corresponding author upon reasonable request.
