# Supplementary material for: MRLC controls apoptotic cell death and functions to regulate epidermal development during planarian regeneration and homeostasis
Source: Cell Prolif. 2023 Jun 25;57(1):e13524. doi: 10.1111/cpr.13524 (PMC10771114; doi:10.1111/cpr.13524)
Supplement: Supplementary file 1 — Data S1. Supporting Information. [file CPR-57-e13524-s001.docx]

**Supplementary Information**

**
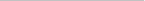
**

SUPPLEMENTARY INFORMATION INDEX

• Supplementary Figure 1–3

• Supplementary Table 1

Fig.S1


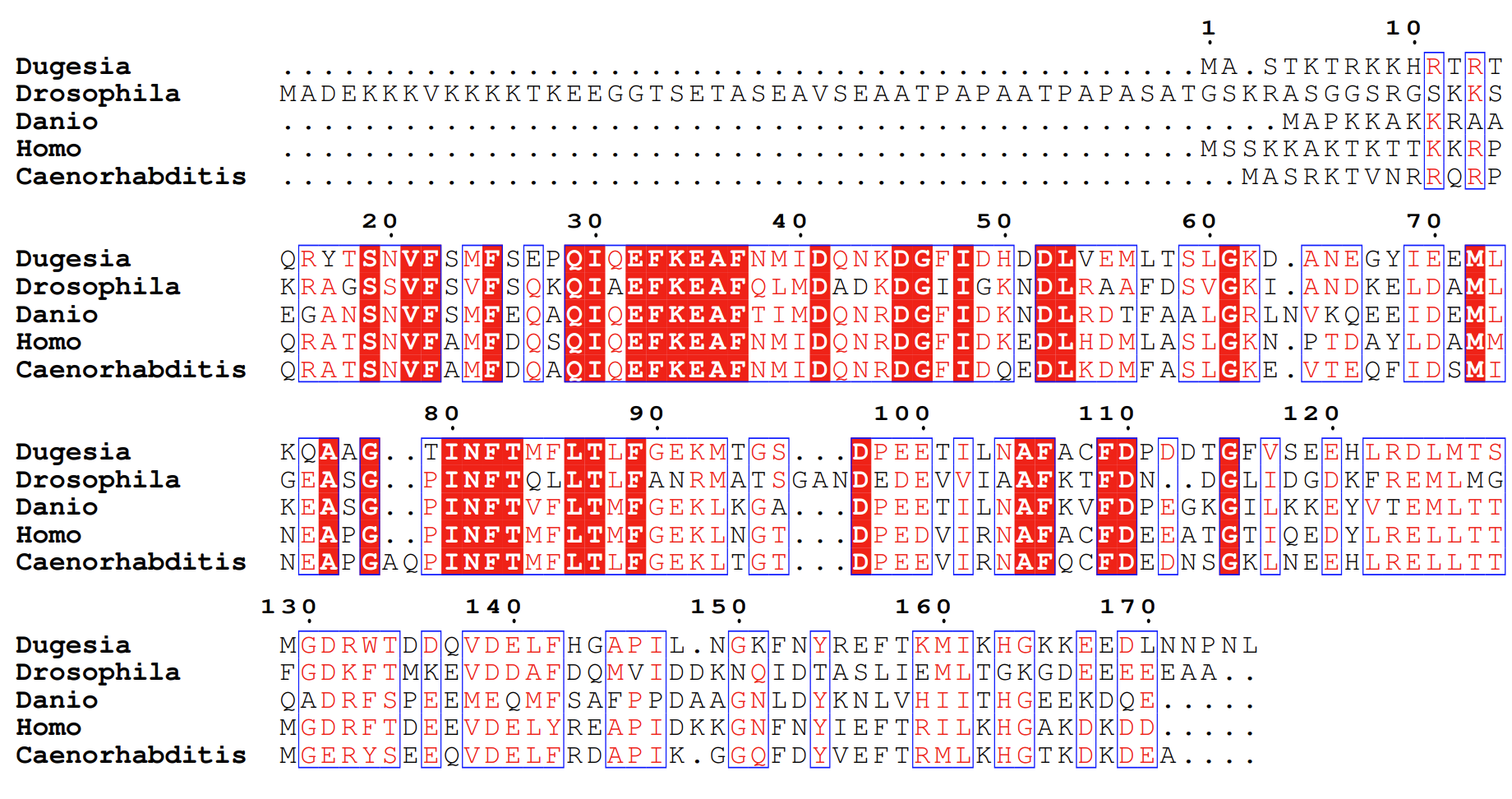


**Fig. S1** Multiple sequence alignment of *MRLC* from *Dugesia japonica*, *Caenorhabditis elegans*, *Danio rerio*, *Drosophila* and *Homo sapiens*. Absolutely conserved residues are in red shaded boxes, and highly conserved residues are colored red and are boxed.

Fig.S2


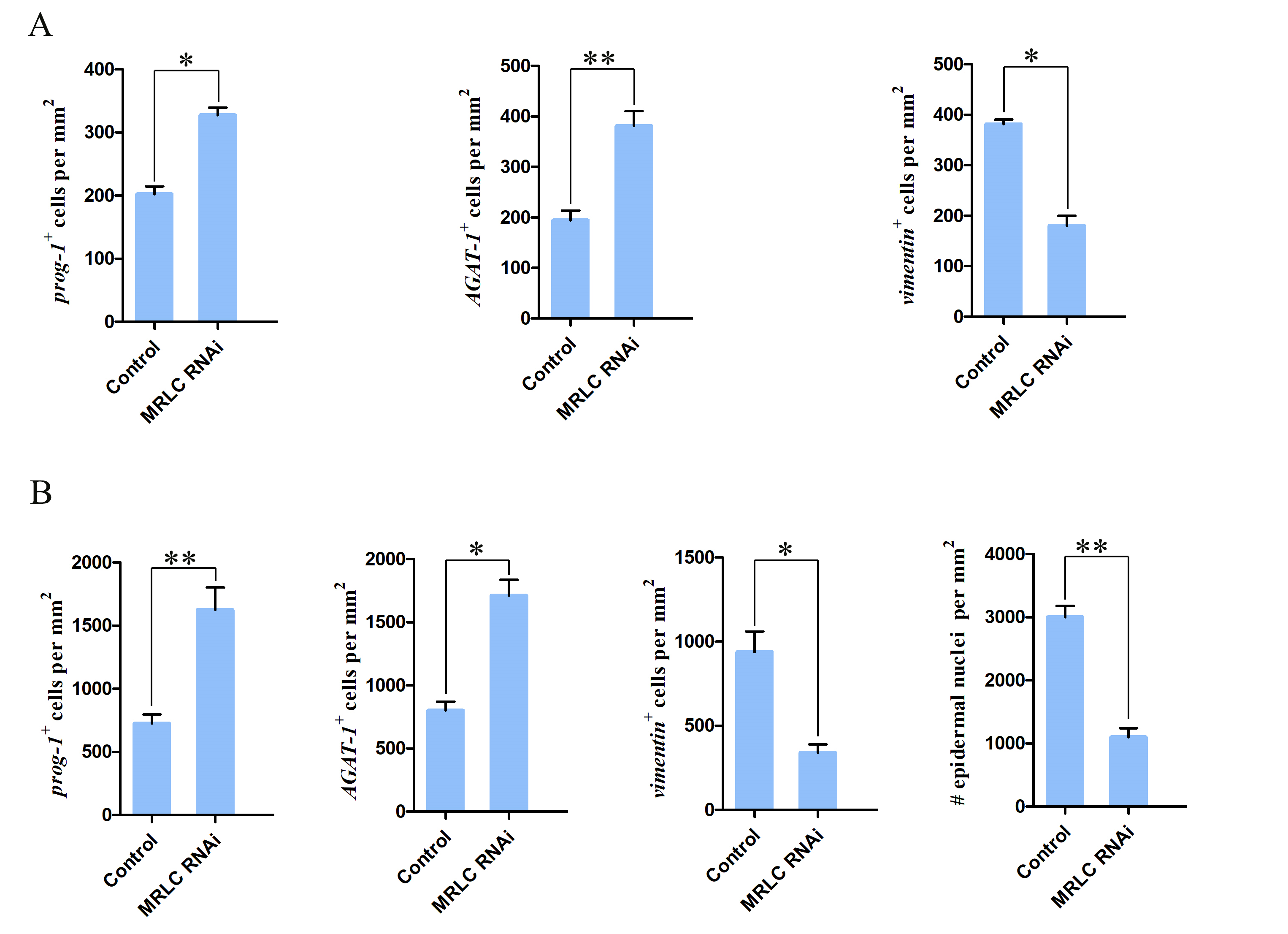


**Fig. S2** Knockdown of *MRLC* impairs epidermal development during planarian regeneration.

(A-B) *Prog-1*^+^early progeny, *AGAT-1*^+^ late progeny and *vimentin*^+^ late progeny and epidermal cell density (DAPI) were quantified at 48 hours and 5dpa in RNAi animals. *p<0.05; **p<0.01.

Fig. S3


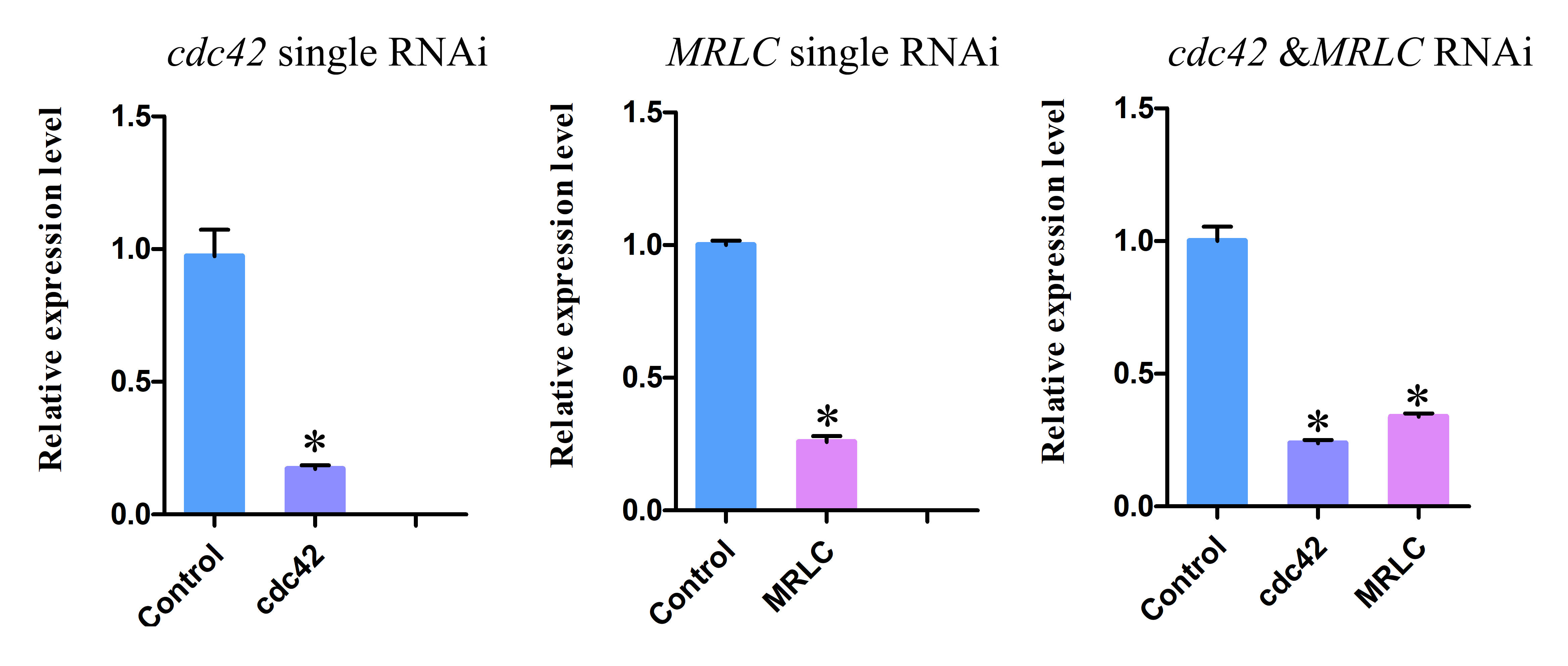


**Fig. S3** Quantification of *cdc42* and *MRLC* transcript levels in single and double RNAis animals were measured through qPCR. Left: the expression level of *cdc42* in *cdc42* RNAi animals; Middle: the expression level of *MRLC* in *MRLC* RNAi animals; Right：the expression level of *cdc42* and *MRLC* in *cdc42* and *MRLC* RNAi animals; *p<0.05.

| Dj*MRLC*- F | CAGATCTTCTTCTTTCTTTCCGTG |
| --- | --- |
| Dj*MRLC*- R | GCTTACATTGAAGAAATGCTCAAGC |
| RNAi-Dj*MRLC*- F | TAATACGACTCACTATAGGG CAGATCTTCTTCTTTCTTTCCGTG |
| RNAi-Dj*MRLC*- R | TAATACGACTCACTATAGGG GCTTACATTGAAGAAATGCTCAAGC |
| insitu- Dj*MRLC*-F | ATGGCATCTACAAAGACAAGAAAGAAG |
| insitu- Dj*MRLC*-R | TAATACGACTCACTATAGGG GCATTTCTTCTATATAACCTTCATTAGC |
| qPCR - Dj*MRLC*-F | ATGGCATCTACAAAGACAAG |
| qPCR - Dj*MRLC*-R | CCGAGTGAAGTTAACATTTC |
| qPCR-Dj *prog-1*F | GAGGTGATGACAAATCGAAGGA |
| qPCR- Dj*prog-1* R | CCTCCTCACCTTCACTTTCTTT |
| qPCR-Dj*piwi-1*F | GAATGAGAGAAGGTCCCAGTTT |
| qPCR-Dj*piwi-1*R | GAAGCCGTTCCATCATCATTTG |
| qPCR-Dj*AGAT-1* F | ACCGATGTGTCGAGAAAGTTTA |
| qPCR-Dj*AGAT-1* R | CAGCATCAAACACAGGTTCTTC |
| qPCR-Dj*vimentin-* F | CTAGCATTGGGGATAATGGA |
| qPCR-Dj*vimentin-* R | CATTGAACTCGTTCCAAATCC |
| qPCR-Dj*ef2*- F | CAGGAGTTTGTGTTCAGACAG |
| qPCR-Dj*ef2*- R | CTGTAAACCGGAACCAAAACC |
| qPCR-Dj*collagen*- F | CAAATGGTGGTCGGACGAGT |
| qPCR-Dj*collagen-* R | TTGAATGGCCGTGTCACTGT |
| qPCR-Dj*PC2*- F | CGCTGGATTGCGTATGTTGG |
| qPCR-Dj*PC2-* R | CGGCCTTTGTTAACGCCTTC |
| qPCR-Dj*hnf4*- F | ATGCCGGACCAGAAGGAATG |
| qPCR-Dj*hnf4-* R | ACTGATTCGATCGCGTTCAT |
| qPCR-Dj*LaminB*- F | CGATATGGAAGCTCAATACACT |
| qPCR-Dj*LaminB-* R | GATTCAAGTTCCGACTGTCGATTC |
| RNAi-Dj*cdc42*- F | TAATACGACTCACTATAGGG AGGCCATTGAGTTATCCGAACA |
| RNAi-Dj*cdc42*- R | TAATACGACTCACTATAGGG TATCAATGAACATTTTCGTTTAC |
| qPCR - Dj*cdc42*-F | TGGAGATGGTGCTGTTGGTAA |
| qPCR - Dj*cdc42*-R | TGTTCGGATAACTCAATGGCCT |

Table.S1 PCR primers used in this study.
